# Supplementary material for: A Comprehensive Study of De Novo Mutations on the Protein-Protein Interaction Interfaces Provides New Insights into Developmental Delay
Source: Biomolecules. 2022 Nov 6;12(11):1643. doi: 10.3390/biom12111643 (PMC9687726; doi:10.3390/biom12111643)
Supplement: Supplementary file 1 [file biomolecules-12-01643-s001.zip › Supplementary figures.pdf]

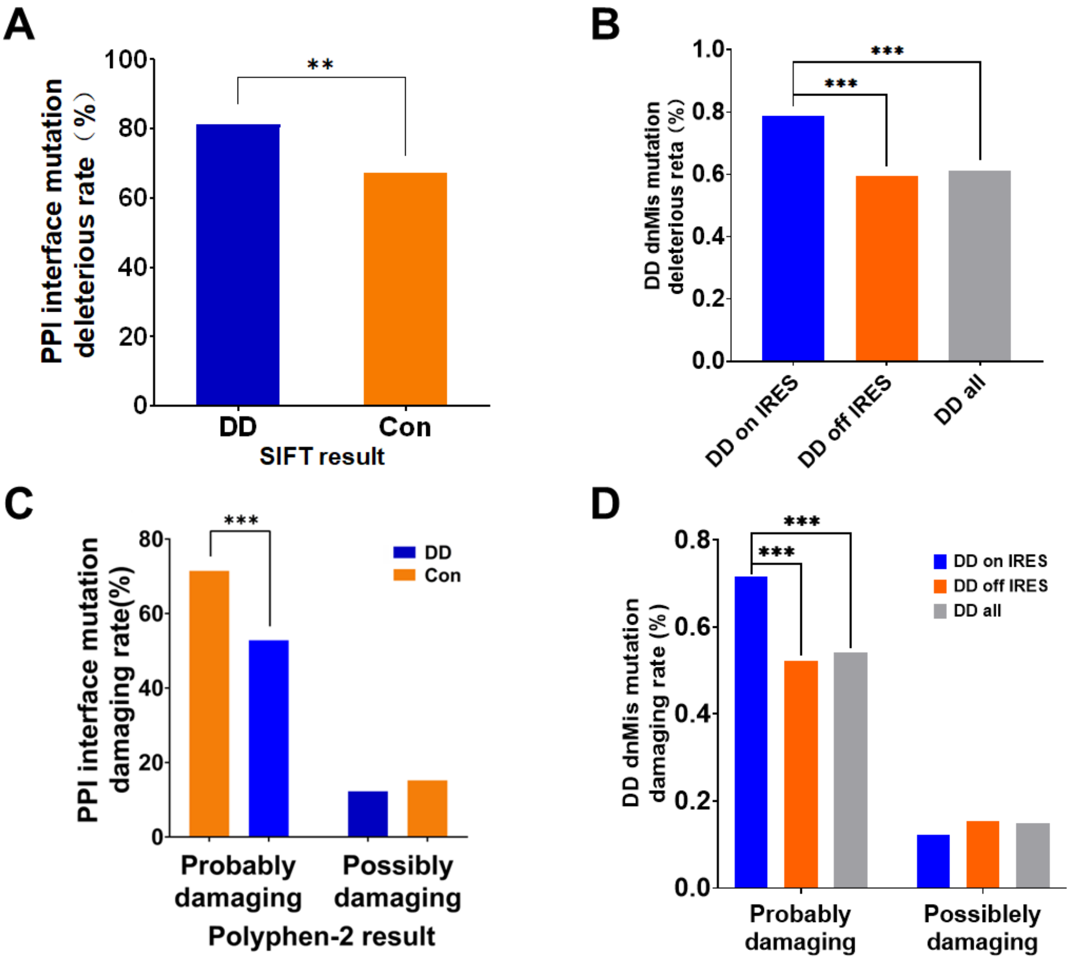

Supplementary Figure S1. PPI interface DNMs enrichment and deleteriousness in DD. A, Deleterious rate of the SIFT prediction toward PPI interface dnMis mutations, if the SIFT prediction score <=0.05, then the mutation will be defined as deleterious. **B**, deleterious rate of the SIFT prediction toward dnMis mutations in DD patients. **C,** Influential rate of the Polyphen-2 prediction toward PPI interface dnMis mutations, if the Polyphen-2 score >=0.956 or >=0.452, then the mutations will be recognized ad probably damaging or possibly damaging respectively. **D**, influential rate of Polyphen-2 prediction toward dnMis mutations in DD patients. IRES represent interface residues.

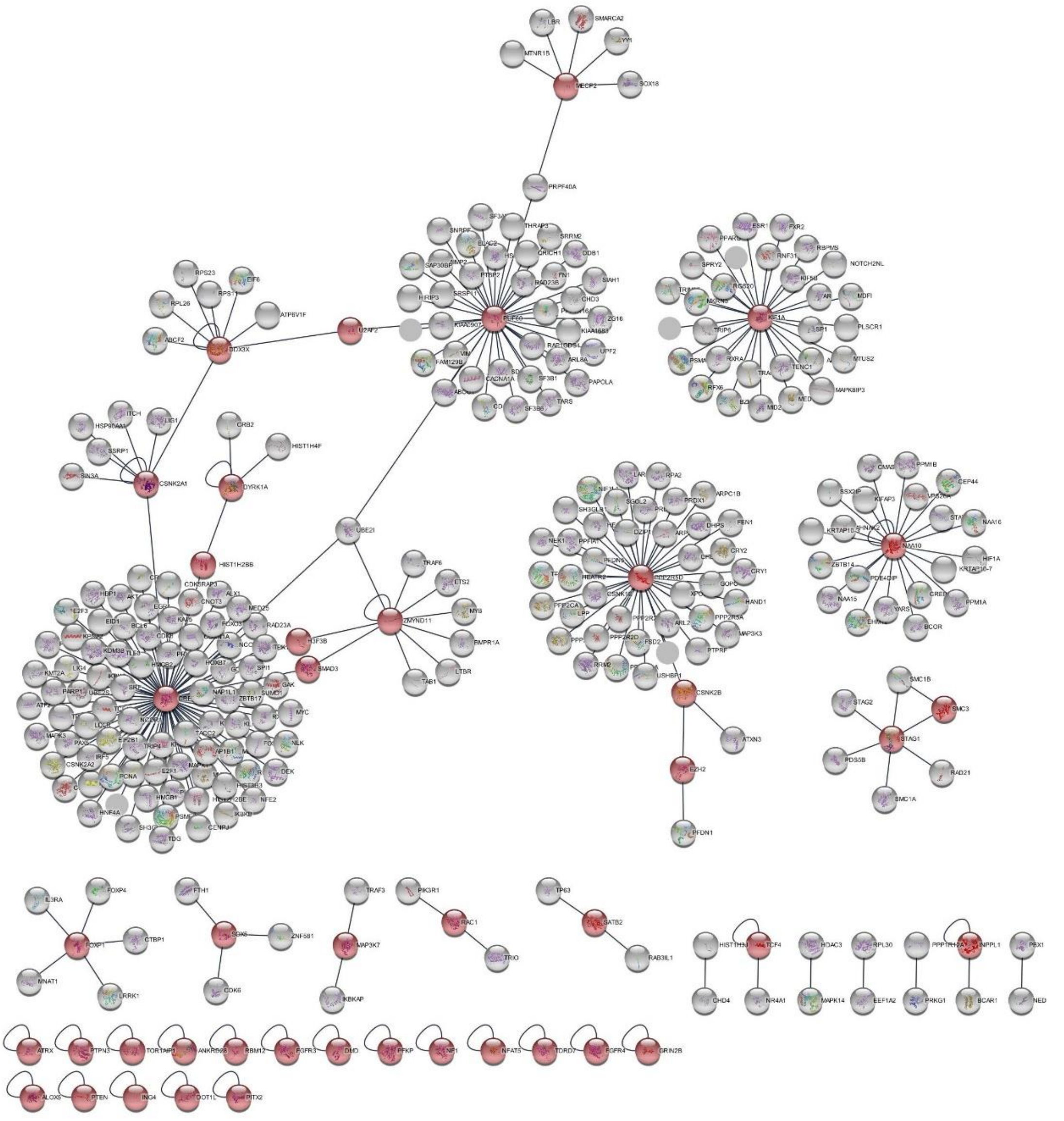

Supplementary Figure S2. PsychiPPI network calculated by a binomial statistical model. The PsychiPPI network was visualized by Cytoscape. Nodes represent protein, and edges represent the interaction between two proteins. The red nodes is the DD candidate proteins that harboring a significantly excess number of its PPI interface mutations

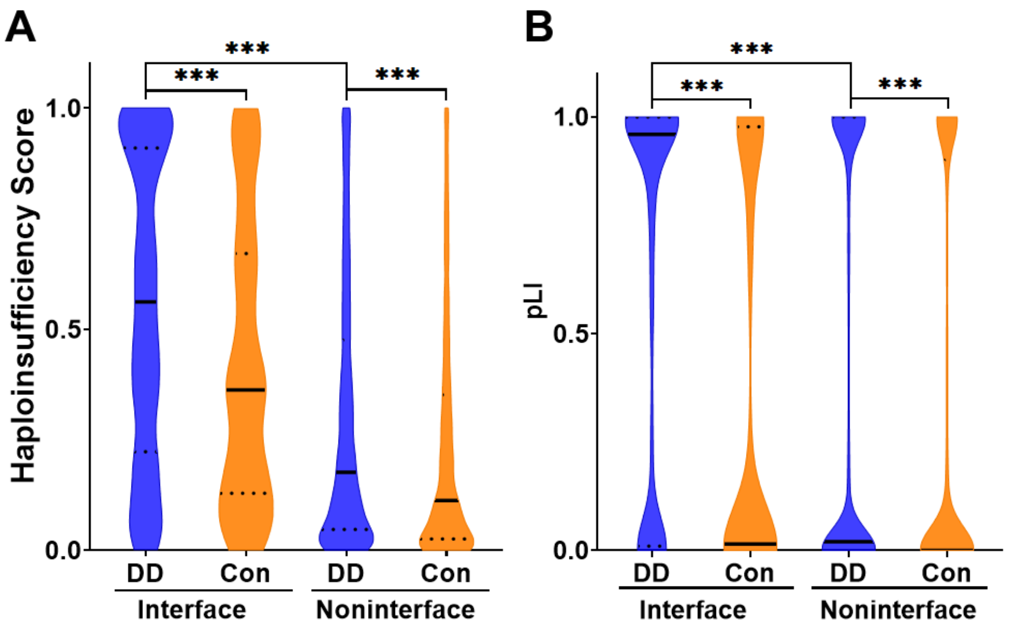

Supplementary Figure S3. variant tolerance distribution of the de novo mutations genes A, dnMis mutations' Haploinsufficiency score of DD and sibling control. **B,** dnMis mutations' pLI score of DD and sibling control. IRES represent interface residues.

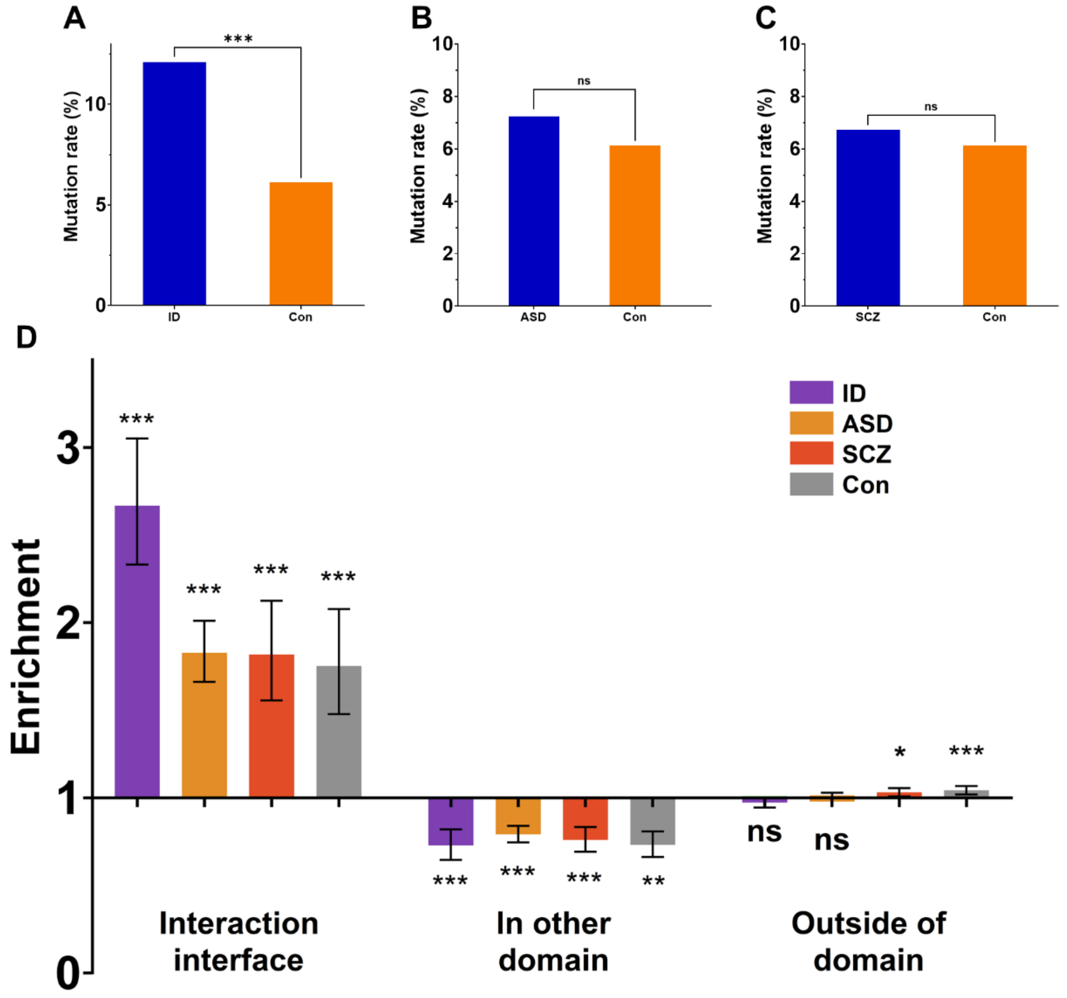

Supplementary Figure S4. PPI interface dnMis mutations enrichment and mutations rate in other neuropsychiatric disorders. A,B,C the PPI interface dnMis mutations rate of intellectual disability(ID), autism spectrum disorders(ASD), and schizophrenia(SCZ) **D,** the PPI interface dnMis mutations enrichment between ID, ASD, SCZ, and sibling control.
